# Putative periodontopathic bacteria and herpesviruses in pregnant women: a case-control study

**DOI:** 10.1038/srep27796

**Published:** 2016-06-15

**Authors:** Haixia Lu, Ce Zhu, Fei Li, Wei Xu, Danying Tao, Xiping Feng

**Affiliations:** 1 Department of Preventive Dentistry, Ninth People’s Hospital, School of Medicine, Shanghai Jiao Tong University, Shanghai Key Laboratory of Stomatology, Shanghai, China; 2 Department of Preventive Dentistry, Shanghai Stomatological Hospital, Shanghai, China

## Abstract

Little is known about herpesvirus and putative periodontopathic bacteria in maternal chronic periodontitis. The present case-control study aimed to explore the potential relationship between putative periodontopathic bacteria and herpesviruses in maternal chronic periodontitis.Saliva samples were collected from 36 pregnant women with chronic periodontitis (cases) and 36 pregnant women with healthy periodontal status (controls). Six putative periodontopathic bacteria (*Porphyromonas gingivalis* [Pg], *Aggregatibacer actinomycetemcomitans* [Aa], *Fusobacterium nucleatum* [Fn], *Prevotella intermedia* [Pi], *Tannerella forsythia* [Tf], and *Treponema denticola* [Td]) and three herpesviruses (Epstein-Barr virus [EBV], human cytomegalovirus [HCMV], and herpes simplex virus [HSV]) were detected. Socio-demographic data and oral health related behaviors, and salivary estradiol and progesterone levels were also collected. The results showed no significant differences in socio-demographic background, oral health related behaviors, and salivary estradiol and progesterone levels between the two groups (all *P* > 0.05). The detection rates of included periodontopathic microorganisms were not significantly different between the two groups (all *P* > 0.05), but the coinfection rate of EBV and Pg was significantly higher in the case group than in the control group (*P* = 0.028). EBV and Pg coinfection may promote the development of chronic periodontitis among pregnant women.

Periodontitis is one of the most common infectious diseases among pregnant women. While pregnancy itself does not cause periodontitis, changes in sex hormone levels during pregnancy may exacerbate the underlying periodontal inflammation^1^. The relationship between maternal periodontitis and pregnancy complications (e.g. premature and low-birth-weight infants) has long been a hot research topic. A recent systematic review indicates that mothers with periodontal disease are more likely to deliver premature and low-birth-weight infants^2^. Periodontitis is not only hazardous to women’s health, but may also bring health risks to newborns. Etiological studies on maternal periodontitis are essential for the effective prevention and treatment of this condition.

As a multifactorial disease, periodontal disease involves an interplay between microbiological, host, and environmental factors^3^. According to conventional etiological studies, specific types of bacteria and their metabolites are believed to trigger periodontal disease, and the interaction between the trigger and host finally results in damage to the periodontal supporting tissues^4^. However, research has demonstrated that these particular types of pathogenic bacteria are not detected in individuals suffering from periodontal disease; or the distribution of these bacteria does not differ significantly between diseased patients and healthy individuals^5^,^6^. The bacterial pathogenesis theory cannot fully explain the clinical features of periodontal disease alone, and the conventional treatments targeting such bacteria have limited roles in preventing periodontal disease^7^. Viruses may also play a role in the pathogenesis of periodontal disease. The herpesvirus has been known to be a pathogenic factor for various periodontal diseases since the 1990s^8^. Slots found that the pathogenicity of the coinfection of herpesvirus and putative periodontopathic bacteria was stronger than the simple sum of these two microbes alone; they concluded that modern periodontitis pathogenesis models should be based on the coinfection of herpesviruses and putative periodontopathic bacteria^9^.

Nowadays, etiological studies of various diseases have been transformed from simple biomedical models to broader multidimensional and multifactorial stereoscopic models, covering biological, behavioral, social, and many other factors. Microorganisms are essential factors in the pathogenesis of periodontal disease during pregnancy; however, their presence alone does not mean that periodontal disease will surely occur during pregnancy. The risk factors for periodontal disease also include socio-demographic factors, oral and systemic health behaviors, diet habits, and others^10^. Therefore, etiological studies of periodontal disease should not be limited to microorganisms as causative agents; instead, many other factors, including behaviors and socio-demographic background, should also be taken into consideration. Although a number of studies have explored the etiology of maternal periodontitis, very rare studies consider the effects of behaviors and socio-demographic background on the maternal periodontitis and balanced these factors between case and control groups.

To our knowledge, no study has described the detection rate of herpesvirus in pregnant women with chronic periodontitis, or its relationship with putative periodontopathic bacteria. In keeping with multifactorial etiological models and modern microbial pathogenesis models of periodontitis, the present case-control study aimed to explore the potential relationship between putative periodontopathic bacteria and herpesviruses in maternal chronic periodontitis. A comparison of their detection rates in saliva was made in pregnant women with chronic periodontitis and those with a healthy periodontal status, after balancing their socio-demographic background, oral health related behaviors, and salivary estradiol and progesterone levels.

## Results

A total of 72 pregnant women (36 for case group and 36 for control group) were enrolled in the present study. Comparisons of the socio-demographic background and oral health behaviors of these two groups are shown in Table 1. No significant difference was found in terms of socio-demographic background including age, gestational age and oral health related behaviors such as tooth brushing and use of dental floss (all *P* > 0.05) between two groups. The hormone levels in the two groups are also shown in Table 1. The progesterone and estradiol levels were not significantly different between the case group and the control group (both *P* > 0.05). Thus, these two groups were well matched.

Comparisons of the periodontal clinical parameters in the two groups are shown in Table 1. VPI, BOP, and CAL were significantly different between the case group and the control group (all P < 0.001), while PD showed no significant difference (P = 0.288).

**Figure 1:**
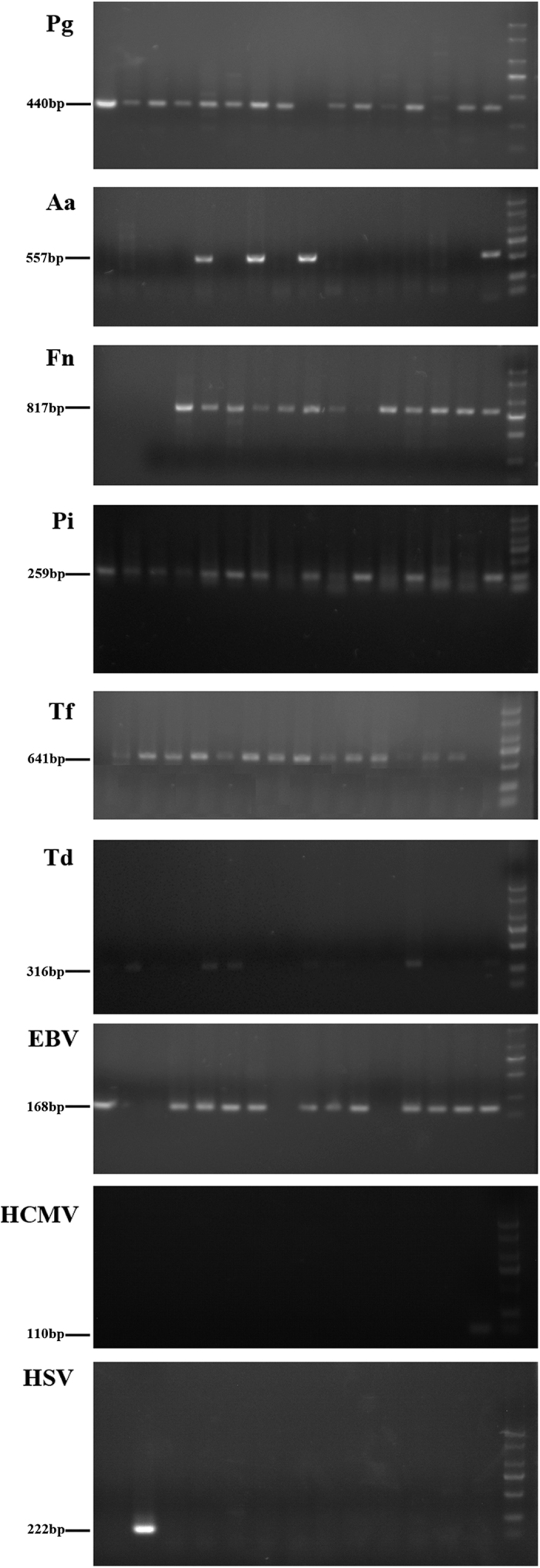
Electrophoresis-positive Lanes of Periodontopathic Microorganisms.

The detection rates for putative periodontopathic bacteria and herpesviruses in the two groups are shown in Table 2. There was no significant difference in the detection rates for putative periodontopathic bacteria and herpesviruses between the case group and the control group (all *P* > 0.05). The gel electrophoresis results are shown in Fig. 1.

The detection rates of putative periodontopathic bacteria and herpesvirus coinfection in the two groups are shown in Table 3. Although EBV + Pg coinfection was significantly different between the two groups (*P* = 0.028), coinfection of other microorganisms showed no significant differences between the two groups. The OR value of EBV + Pg coinfection was 3.00 and the 95% CI was 1.11–8.14.

## Discussion

Slots presented evidence that viruses and bacteria in aggregate can produce a greater pathogenic effect than the sum of the individual agents, and the current model of the pathogenesis of periodontitis should be revisited according to the concept of a herpesviral-bacterial coinfection^9^. While many studies have explored putative periodontopathic bacteria and herpesvirus coinfections in populations, few studies have targeted such coinfections in pregnant women with periodontitis. Our current study is the first case-control study pertaining to periodontopathic bacteria and herpesvirus infections in pregnant women, with an attempt to investigate the potential correlation between periodontopathic bacteria, herpesviruses and maternal periodontitis.

Multidimensional etiological research models need to consider biological, social, behavioral, and many other factors. In the present study, we matched the socio-demographic background and oral health-related behavioral factors between the case and control groups. The progesterone and estrogen levels among these pregnant women were also considered. Accurate study on the microbial pathogenic mechanisms could be conducted based on this well matched case-control study.

Eight members of the herpes family are known to cause human disease. These are Epstein-Barr virus (EBV), human cytomegalovirus (HCMV), herpes simplex virus 1 and 2 (HSV-1, HSV-2), varicella zoster virus (VZV), human herpesvirus 6 (HHV-6), human herpesvirus 7 (HHV-7), and human herpesvirus 8 (HHV-8). In the past decade, a number of studies have been conducted to investigate the influence of herpesviruses on the clinical characteristics of chronic periodontitis^11^,^12^,^13^. A systematic review^14^ indicated that EBV, HCMV, and HSV might be significantly associated with increased risk of chronic periodontitis. Thus, with limited resources, detection rate of EBV, HCMV, and HSV were investigated in the present study.

In our case group, the detection rates of Pg, Aa, Fn, Pi, Tf, and Td were 86.1%, 83.3%, 83.3%, 69.4%, 91.7%, and 55.6%, respectively, which were consistent with the findings of Ashimoto *et al.*^15^. These detection rates were higher than Tellapragada *et al.*’s study^16^ which also investigated prevalence of putative periodontopathic bacteria among pregnant women with periodontitis in India. Since the mean age and gestational age between the present study and study conducted in India were similar (age: 26.9 ± 3.6 *vs.* 26 ± 3.4 years; Gestational age: 18.3 ± 7.5 *vs.* 18 ± 3.5 weeks), the possible explanations for the different detection rates were due to different ethnic group and diagnostic criteria for chronic periodontitis. Distinct technology and primers to detect the putative periodontopathic bacteria may also contribute to the different detection rates. As to the three herpesviruses, the detection rate of EBV in the general population with chronic periodontitis is about 50%^17^,^18^; by contrast, the detection rate of EBV was 86.1% in our case group, which was higher than was reported in previous studies. Eres *et al.*^19^ found that the detection rate of EBV was 38.6% in pregnant women with chronic gingivitis; markedly lower than the rate in our case group. The detection rates of HCMV were dramatically different among the general population with chronic periodontitis^13^,^17^,^18^,^20^; in our case group, the detection rate of HCMV was 8.3%, which was higher than the findings in research investigating the general population with chronic periodontitis conducted by Rotola *et al.* (0%)^18^, but markedly lower than those in similar studies performed by Imbronito *et al.* (50%)^17^ and Wu *et al.* (79.0%)^20^. While Eres *et al.*^19^ found that the detection rate of HCMV was 14.3% in pregnant women with chronic gingivitis; slightly higher than the rate in our case group. The detection rate of HSV in our case group was 5.6%, which was basically consistent with the findings of research conducted by Saygun *et al.* (6.7%)^13^, but markedly lower than a study performed by Imbronito *et al.* (40%)^17^. Different detection rates of EBV, HCMV and HSV were observed among various studies. There studies varied widely on the study participants characteristics (population, ethnicity, age and gender), sample types (saliva, subgingival plaque, biopsy, and gingival crevicular fluid), and detection methods (PCR, real-time PCR, and nested-PCR). All of these diversities may be related to the variation of herpesviruse detection rate among various studies.

The specific pathogenic mechanisms of putative periodontopathic bacteria and herpesvirus coinfection remain unclear. As an infectious disease, the progression of periodontitis is associated with the accumulation of immune cells^21^. Studies suggested that herpesviruses can adjust to immune mechanisms and immune responses^22^,^23^. Herpesviruses can decrease the ability of periodontal tissues to resist bacterial invasion by altering structural cells or host defense cells of the periodontium^24^,^25^. In our current study, the detection rates of six putative periodontopathic bacteria and three herpesviruses showed no significant difference between the case group and the control group; but the detection rate of EBV and Pg coinfection significantly differed between the two groups, consistent with the findings of Saygun *et al.*^26^. The development of periodontitis is a sequential infectious process that proceeds from bacteria to herpesvirus to bacteria^27^. Initially, bacteria in the dental biofilm such as Pg induce gingivitis, which permits latent herpesviruses such as EBV, embedded in the DNA of macrophages, T lymphocytes and B lymphocytes, to infiltrate the periodontium^9^. Imai *et al.*^28^ indicated that Pg has the potential to trigger EBV by increasing the activity of the BZLF1 gene, which encodes the key protein for the transition from latency to the lytic replication cycle. Hormonal changes during pregnancy, as one of herpesvirus-activating factors (other factors like drug-induced immunosuppression, unusual and prolonged emotional stress, physical trauma, etc), may re-activation of the latent EBV spontaneously or during periods of decreased host defense^29^. In response to the active herpesvirus infection, the host elicits a robust T-cell-mediated immune response, comprised primarily of CD8^+^ T cells. To counteract the hostile host environment, herpesviruses in turn execute strategies to down-regulate antiviral host defenses. Herpesviruses evade immune responses by disintegrating components of the major histocompatibility complex and interfering with antigen presentation^30^. The encounter between antiviral host defenses and virally mediated anti-host responses results in a major release of pro-inflammatory cytokines that potentially activate osteoclasts and to impair antibody-mediated host defenses against exogenous-like bacterias, such as Pg^31^. The ensuing increase in pathogenic bacteria leads to additional mechanisms of periodontal tissue destruction.

Several research limitations should be acknowledged in the present case-control study. Firstly, there are only 72 participants in the present study (36 for case group and 36 for control group). According to the summary table from the Meta-analysis based on the case-control studies which investigated the relationships between herpesvirusus and chronic periodontitis^14^, the sample size in the case groups ranged from 13 to 143. Moreover, 7 out of 12 studies included the number of participants between 20 and 40. Based on this perspective, the sample size in the present study is considered to be moderate. Meanwhile, it is the first study comparing the detection rates of six putative periodontopathic bacteria and three herpesviruses among pregnant women with or without chronic periodontitis. Secondly, the gestational age was not even distributed within each group; for the case and control group, 16.7% and 8.3% were in the first trimester, 83.3% and 80.6% were in the second trimester, 0% and 11.1% were in their third trimester. These may lead to a possibility of selection bias. Thirdly, PCR, nested PCR, viral culture and real-time PCR were adopted to detect periodontal herpesviruses. However, studies and review indicated that nested PCR would be the most sensitive and specific method for detection of periodontal herpesviruses^9^,^32^,^33^. As to the study samples, various samples (including saliva, subgingival plaque, biopsy, and gingival crevicular fluid) were collected to detect the periodontal herpesviruses among previous studies. Biopsy specimen is considered to be a superior sample for detection of periodontal herpesviruses because more herperviruses specimen is found in biopsy specimen than in other samples^34^. However, it was difficult to collect biopsy specimens from pregnant women and saliva was collected as the sample to detect herpesviruses. The detection rate of herpesviruses in saliva might be lower than the actual infection rate of herpesviruses that could have been detected in biopsy specimens. However, salivary biomolecules can aid in the diagnosis of a variety of cancers, hereditary disorders, hormonal irregularities, nicotine dependence and pathogenic viruses and bacterias^35^,^36^. Non-stimulated salivary samples can be collected by any health professionals, by the individuals themselves or by parents for young children. Collection of non-stimulated salivary samples is painless, non-invasive, time-saving and involves virtually no health or safety issues, particularly for the pregnant women. Salivary sample, as used to test the periodontopathic bacteria, is based on theory that whole saliva and periodontal lesions tend to harbor similar relative levels of periodontal pathogens^37^. Umeda *et al.*^38^ demonstrated a statistical relationship between the presence of periodontopathic, including Pg, Pi, and Td, in whole saliva and in periodontal pocket samples.

The current treatment and prevention of periodontal diseases is entirely on the concept of bacterial pathogenesis theory. However, if a biological link is established between herpesviruses and periodontal diseases, the conventional therapy aiming at bacteria might be inefficient. Periodontal treatment employing both antiviral agents and antibacterial debridement might be more effective in treating periodontal diseases. Sunde *et al.*^39^ treated a patient with refractory periodontitis and high EBV levels using valacyclovir hydrochloride, resulting in marked improvement of the patient’s periodontal situation. A low dose of oral interferon has been found to reduce gingival inflammation in a canine model^40^. The concept of herpesviral-bacterial synergism in periodontitis implies that vaccination against herpesviruses can also contribute to the control of periodontopathic bacteria^9^. Future management of periodontal diseases may benefit from anti-herpesviral immunotherapeutics: either prophylactic vaccines, which utilize the immune system of healthy subjects to prevent infection with disease-causing viruses; or therapeutic vaccines, which stimulate the immune system into combating existing viruses and disease. Periodontal treatment that includes both antibacterial debridement and antiviral agents or vaccines might be more effective in treating the intractable and recurrent periodontitis.

In summary, although the present study could not confirm a causal relationship between herpes virus and periodontopathogenic -bacteria coinfection in maternal periodontitis, it did suggest that EBV and Pg coinfection may promote the development of chronic periodontitis in pregnant women. Nevertheless, the specific mechanisms of the interaction between herpesviruses and putative periodontopathic bacteria require further investigation.

## Methods

### Participants

A case-control study was conducted of pregnant women in all stages of pregnancy in Shanghai, China. In Shanghai, 17 maternal and child care service centres at a county level have been established. Among these centres, 11 are located in urban districts and 6 are found in rural counties. With limited resources, three centres (two urban and one rural maternal and child care service centres) were randomly selected. In each selected care service centre, pregnant women who attended antenatal checkup and were eligiable for inclusion and exclusion criteria were consecutively recruited from October 2012 to March 2013. The study conformed to STROBE guidelines. Ethical approval was obtained from the Institutional Review Board of the Ninth People’s Hospital, Shanghai Jiao Tong University School of Medicine prior to the implementation of the study and the study was carried out in accordance with relevant guidelines. Written informed consent was obtained from the participants, as appropriate.

Participants in the case group were pregnant women with chronic periodontitis at any gestational stage. The diagnostic criteria for chronic periodontitis were as follows: the presence of four or more teeth showing at least one site with a probing depth (PD) ≥4 mm and clinical attachment level (CAL) ≥4 mm at the same site; and presence of bleeding on probing (BOP)^41^. Other inclusion criteria were: a) aged 20–40 years; and b) retaining at least 20 natural permanent teeth (except the third molar). The exclusion criteria were: a) systemic disease; b) periodontal therapy within the past 3 months; c) use of antibiotics within the past 3 months; and d) obvious abnormal occlusion.

In the control group, only pregnant women with healthy periodontal status were enrolled. The diagnostic criteria for healthy periodontal status were as follows: no periodontitis diagnosed for all teeth and BOP rate of less than 25%. The other inclusion and exclusion criteria were the same as in the case group.

### Periodontal Examination

Periodontal examinations were carried out for all participants in each maternal and child care service center. The data were recorded on a clinical record form with a complete clinical and periodontal description of all the teeth excluding third molars. The visible plaque index (VPI) was assessed as the percentage of surfaces with plaque on two surfaces per tooth using the dichotomous plaque index (presence or absence of plaque). Probing depth (PD: measurement from the gingival margin to the total probing depth) and clinical attachment level (CAL: measurement from the cemento-enamel junction to the total probing depth) were evaluated at six sites per tooth (mesiobuccal, buccal, distobuccal, distolingual, lingual and mesiolingual). Bleeding on probing (BOP) was assessed at the same time as PD was measured, using a dichotomous index (presence or absence of bleeding), and was expressed as the percentage of surfaces showing bleeding. The participants were examined by one trained and calibrated examiner who used a disposable mouth mirror attached to an intraoral LED light and lightweight Community Periodontal Index (CPI) probes with a ball-tip end diameter of 0.5 mm. Approximately 10% of these participants were then re-examined to monitor inter-examiner reproducibility. As measured using Kappa statistics, the inter-examiner reliability of VPI, BOP, PD and CAL were 0.86 (95% CI:0.81–0.91), 0.72 (95% CI: 0.66–0.78), 0.75 (95% CI: 0.71–0.79) and 0.70 (95% CI: 0.64–0.76), respectively.

### Questionnaires

After the clinical examinations, the participants were instructed to complete a structured questionnaire to obtain information about their socio-demographic background (including age, birth place, location, marital status, gestational age, monthly household income, educational level and dental insurance coverage) and oral health behaviors (including daily tooth brushing frequency, use of dental floss and mouthrinse, and dental visit pattern).

### Saliva Sample Collection and Preparation

Non-stimulated whole saliva (2 ml) was collected from each participant before the periodontal examination and stored in an icebox, which was transported to the laboratory for processing within 4 hours. Aliquots of 0.5 ml of the saliva samples were then centrifuged at 16,000 g for 5 min. The remaining precipitate was repeatedly rinsed with PBS buffer at least five times, and then stored at −20 °C for DNA extraction. Another 0.5 ml of the saliva sample was centrifuged at 4000 g for 10 min; and the supernatants were stored at −20 °C for the measurement of hormone levels.

### Measurement of Hormone Levels

After the supernatants were thawed, the progesterone level was determined using a saliva progesterone kit (Demeditec Diagnostics, Kiel-Wellsee, Germany). Two duplicate wells were used per sample (unit: pg/ml). After the salivary supernatant was diluted 1/100 with normal saline, the estradiol level was measured using a salivary estradiol ELISA kit (Demeditec). Two duplicate wells were used per sample (unit: pg/ml).

### PCR Procedures

Extraction of saliva genomic DNA was performed using QIAamp DNA Mini kit (Qiagen) in accordance with the instruction manual. Six putative periodontopathic bacteria (*Porphyromonas gingivalis* [Pg], *Aggregatibacter actinomycetemcomitans* [Aa], *Fusobacterium nucleatum* [Fn], *Prevotella intermedia* [Pi], *Tannerella forsythia* [Tf], and *Treponema denticola* [Td]) were detected using 16 S rDNA-based polymerase chain reaction (PCR). The primers of these six microorganisms were determined based on relevant literature^15^,^42^ and NCBI Basic Local Alignment Search Tool (BLAST) and then synthesized by Shanghai Sangon. Meanwhile, three herpesviruses (Epstein-Barr virus [EBV], human cytomegalovirus [HCMV], and herpes simplex virus [HSV]) were detected using nested PCR. The primers of these three viruses were determined based on relevant literature^12^,^18^,^43^ and NCBI BLAST and then synthesized by Shanghai Sangon. Primer sequences are shown in Table 4.

PCR amplification was performed on a PCR machine (Bio-Rad). The 25-μL PCR system consisted of a 2 μL DNA template (0.5 μL inner DNA template) and 2 × 12.5 μl UTaq PCR Mix. The final concentrations of the upstream and downstream primers were 0.4 μmol/L, and deionized water was added to reach 25 μl. The reaction conditions of Pg, Aa, Fn, and Tf were as follows: initial denaturation at 94 °C for 3 min, followed by 30 cycles of denaturation at 94 °C for 30 s, annealing at 55 °C for 30 s, and extension at 72 °C for 30 s, with a last extension at 72 °C for 5 min. The reaction conditions of Pi and Td were as follows: initial denaturation at 94 °C for 3 min, followed by 30 cycles of denaturation at 94 °C for 30 s, annealing at 55 °C for 30 s, and extension at 72 °C for 20 s, with a last extension at 72 °C for 5 min. The reaction conditions of the inner and outer primers of EBV were as follows: initial denaturation at 94 °C for 3 min, followed by 35 cycles of denaturation at 94 °C for 30 s, annealing at 63 °C for 15 s, and extension at 72 °C for 30 s, with a last extension at 72 °C for 1 min. The reaction conditions of the outer primer of HCMV were as follows: initial denaturation at 94 °C for 5 min, followed by 35 cycles of denaturation at 94 °C for 30 s, annealing at 57 °C for 30 s, and extension at 72 °C for 30 s, with a last extension at 72 °C for 3 min. The reaction conditions of the inner primer of HCMV were as follows: initial denaturation at 94 °C for 5 min, followed by 25 cycles of denaturation at 94 °C for 30 s, annealing at 59 °C for 30 s, and extension at 72 °C for 30 s, with a last extension at 72 °C for 3 min. The reaction conditions of the outer primer of HSV were as follows: initial denaturation at 94 °C for 1 min, followed by 30 cycles of denaturation at 94 °C for 30 s, annealing at 60 °C for 1 min, and extension at 72 °C for 30 s, with a last extension at 72 °C for 1 min. The reaction conditions of the inner primer of HSV were as follows: initial denaturation at 94 °C for 1 min, followed by 30 cycles of denaturation at 94 °C for 1 min, annealing at 55 °C for 1 min, and extension at 72 °C for 30 s, with a last extension at 72 °C for 1 min. For each batch of samples, negative control was set for PCR amplification using sterile deionized water. Duplicate detection was performed for 20% of samples; if the results were inconsistent, a third test was performed. Detection of PCR amplification products: 5 μl PCR products were visualized by 1.5% agarose gel electrophoresis. The marker was DL 2000. The results were observed under UV light.

### Statistical Analysis

Statistical analysis was performed using SPSS version 23.0 (IBM, Armonk, NY, USA). The descriptive statistics (mean, standard deviation or percentage) of all variables were recorded. Chi-square tests or two sample t tests were performed to compare differences in socio-demographics, oral health-related behaviors, hormone levels, periodontal clinical parameters and prevalence of periodontopathic microorganisms between the case group and the control group. *P* < 0.05 was considered statistically significant and all *P*-values were two-sided.

## Additional Information

**How to cite this article**: Lu, H. *et al.* Putative periodontopathic bacteria and herpesviruses in pregnant women: a case-control study. *Sci. Rep.* **6**, 27796; doi: 10.1038/srep27796 (2016).

## Figures and Tables

**Table 1:** Comparisons of the Socio-demographic Background, Oral Health Behaviors, Hormone Levels and Periodontal Clinical Parameters Between Case and Control Groups.

Socio-demographic Background, Oral Health Behaviors,Hormone Levels and Periodontal Clinical Parameters		Case group(n = 36)	Control group(n = 36)	
		N (%)			N (%)	*P* value
Age	Mean ± SD	27.2 ± 4.1	26.9 ± 3.6	0.714
Gestational age (weeks)	Mean ± SD	19.2 ± 7.8	18.3 ± 7.5	0.624
Marital status	Married	34(94.4)	33(91.7)	1.000
	Unmarried	2(5.6)	3(8.3)	
Birth place	Shanghai	9(25.0)	10(27.8)	0.789
	Other places	27(75.0)	26(72.2)	
Location	Urban	5(13.9)	5(13.9)	1.000
	Suburb	31(86.1)	31(86.1)	
Previous births	None	23(63.9)	25(69.4)	0.617
	One or more	13(36.1)	11(30.6)	
Education level	High school or below	23(63.9)	23(63.9)	0.183
	College	13(36.1)	10(27.8)	
	Master degree or above	0(0.0)	3(8.3)	
Dental insurance coverage	Yes	19(52.8)	18(50.0)	0.814
	Not	17(47.2)	18(50.0)	
Monthly household income	RMB 9000 or below	29(80.6)	33(91.7)	0.173
	Above RMB 9000	7(19.4)	3(8.3)	
Daily tooth brushing frequency	1 or 0	7(19.4)	10(27.8)	0.625
	2	27(75.0)	25(69.4)	
	3 or more	2(5.6)	1(2.8)	
Daily use of dental floss	Yes	1(2.8)	1(2.8)	1.000
	No	35(97.2)	35(97.2)	
Daily use of mouthrinse	Yes	15(41.7)	12(33.3)	0.465
	No	21(58.3)	24(66.7)	
			
Routine dental visits	Yes	0(0.0)	3(8.3)	0.238
	No	36(100.0)	33(91.7)	
Smoking	Current smoker	0(0.0)	0(0.0)	N/A
	Non-smoker	36(100.0)	36(100.0)	
Alcohol-drinking	Yes	0(0.0)	0(0.0)	N/A
	No	36(100.0)	36(100.0)	
Progesterone	Mean ± SD	2262.6 ± 1223.9	2406.7 ± 944.8	0.578
Estradiol	Mean ± SD	2309.8 ± 1508.7	1919.4 ± 1242.4	0.235
VPI( = 1)%^a^	Mean ± SD	58.3 ± 18.8	36.3 ± 18.8	<0.001
BOP( = 1)%^a^	Mean ± SD	58.9 ± 23.4	15.9 ± 4.9	<0.001
PD(≥4 mm) %	Mean ± SD	27.5 ± 17.0	23.1 ± 7.6	0.288
CAL(≥4 mm)%*	Mean ± SD	11.2 ± 13.4	1.8 ± 3.7	<0.001

N/A, not applicable.

^a^*P *< 0.05.

**Table 2:** Detection Results of Putative Periodontopathic Bacteria and Herpesviruses in Two Groups.

Periodontapothicmicroorganisms	Case group (n = 36)	Control group (n = 36)	*P* value
	N (%)	N (%)	
**Pg**			0.052
Detected	31(86.1)	24(66.7)	
Not detected	5(13.9)	12(33.3)	
**Aa**			0.743
Detected	30(83.3)	31(86.1)	
Not detected	6(16.7)	5(13.9)	
**Fn**			0.496
Detected	30(83.3)	32(88.9)	
Not detected	6(16.7)	4(11.1)	
**Pi**			0.617
Detected	25(69.4)	23(63.9)	
Not detected	11(30.6)	13(36.1)	
**Tf**			1.000
Detected	33(91.7)	33(91.7)	
Not detected	3(8.3)	3(8.3)	
**Td**			0.238
Detected	20(55.6)	15(41.7)	
Not detected	16(41.7)	21(58.3)	
**EBV**			0.089
Detected	31(86.1)	25(69.4)	
Not detected	5(13.9)	11(30.6)	
**HCMV**			0.607
Detected	3(8.3)	1(2.8)	
Not detected	33(91.7)	35(97.2)	
**HSV**			1.000
Detected	2(5.6)	1(2.8)	
Not detected	34(94.4)	35(97.2)	

N/A, not applicable.

^a^P < 0.05.

**Table 3:** Comparisons of Putative Periodontapothic Bacteria and Herpesviruses Coinfections in Two Groups.

Coinfection	Case group (n = 36)	Control group (n = 36)	*P* value
	Positive rate (%)	Positive rate (%)	
EBV + Pg^a^	75.0	50.0	0.028
EBV + Aa	13.9	11.1	1.000
EBV + Fn	72.2	69.4	0.795
EBV + Pi	61.1	41.7	0.099
EBV + Tf	77.8	61.1	0.125
EBV + Td	58.3	55.6	0.812
HCMV + Pg	5.6	2.8	1.000
HCMV + Aa	5.6	0.0	0.473
HCMV + Fn	8.3	2.8	0.607
HCMV + Pi	8.3	0.0	0.238
HCMV + Tf	8.3	2.8	0.607
HCMV + Td	5.6	0.0	0.473
HSV + Pg	5.6	2.8	1.000
HSV + Aa	0.0	2.8	1.000
HSV + Fn	5.6	2.8	1.000
HSV + Pi	5.6	2.8	1.000
HSV + Tf	5.6	2.8	1.000
HSV + Td	5.6	2.8	1.000

^a^*P *< 0.05.

**Table 4:** Primer Sequences of Six Putative Periodontopathic Bacteria and Three Herpesviruses.

Periodontapothicmicroorganisms	Primer sequence (5′-3′)	Amplified fragment length (bp)
Pg	AGGCAGCTTGCCATACTGCG ACTGTTAGCAACTACCGATGT	404
Aa	AAACCCATCTCTGAGTTCTTCTTC ATGCCAACTTGACGTTAAAT	557
Fn	AGGGCATCCTAGAATTAT GGGACACTGAAACATCTCTGTCTCA	817
Pi	CGTGGACCAAAGATTCATCGGTGGA CCGCTTTACTCCCCAACAAA	259
Tf	GCGTATGTAACCTGCCCGCA TGCTTCAGTGTCAGTTATACCT	641
Td	TAATACCGAATGTGCTCATTTACAT TCAAAGAAGCATTCCCTCTTCTTCTTA	316
EBV outer primer	GCGGGTGGAGGGAAAGG GTCAGCCAAGGGACGCG	N/A
EBV inner primer	AGGCTGCCCACCCTGAGGAT GCCACCTGGCAGCCCTAAAG	168
HCMV outer primer	AAGCGGCCTCTGATAACCAAGCC AGCACCATCCTCCTCTTCCTCTGG	N/A
HCMV inner primer	AGTGTGGATGACCTACGGGCCATCG GGTGACACCAGAGAATCAGAGGAGC	110
HSV outer primer	GGGCCAGGCGCTTGTTGGTGTA TACATCGGCGTCATCTGCGGGG	N/A
HSV inner primer	CAGTTCGGCGGTGCGGACAAA GCGTTTATCAACCGCACCTCC	222

N/A, not applicable.
